# Supplementary material for: Magnetic field sensing for 2D fault localization in underground power cables
Source: PLoS One. 2025 Oct 16;20(10):e0334515. doi: 10.1371/journal.pone.0334515 (PMC12530618; doi:10.1371/journal.pone.0334515)
Supplement: Attachment — Submitted filename: Response to reviewers.docx [file pone.0334515.s001.docx]

**Original Manuscript ID:** PONE-D-25-36102

**Original Article Title: “**Magnetic Field Sensing for 2D Fault Localization in Underground Power Cables”

**To:** Editor Plos One

**Re:** Response to reviewers

Dear Editor,

Thank you for allowing a revision of our manuscript, with an opportunity to address the reviewers’ comments.

We are uploading (a) our point-by-point response to the comments (below) (Response to Reviewers), (b) an updated manuscript with blue text indicating changes (as “Revised Manuscript with Track Changes*”*), and (c) a clean updated manuscript without highlights (“Manuscript”*).*

Best regards,

Hamid et al.

**Reviewer#1, Concern # 1:**  The opinion of this reviewer is that this manuscript proposes a new method for locating various types of faults in underground power cables. However, none of the three faults considered has an experimental background that could be given for at least one of the three faults. Specifically, this study lacks an adequate experimental background. Finally, any new method must be validated using relevant experimental data.

**Author response:** We sincerely thank the reviewer for this valuable comment. Experimental validation of the proposed method is added in revised manuscript.

**Author action:** The proof from the manuscript is given below:

**Reviewer#1, Concern # 2:** There are similar methods in the literature that the Authors did not use in the literature review. Why? For example, the following publications: 10.1109/TMAG.2013.2297195, 10.1061/41202(423)242, etc.

**Author response:** We sincerely thank the reviewer for pointing out these valuable references. The suggested publications [10.1109/TMAG.2013.2297195, 10.1061/41202(423)242] have now been carefully studied and incorporated into the revised manuscript.

**Author action:** The proof from the manuscript is given below:

**Reviewer#1, Concern # 3:** Novelties and scientific contributions must be contrasted to the relevant state-of-the-art and highlighted in an appropriate manner.

**Author response:** We agree with the honorable reviewer and updated the revised manuscript accordingly.

**Author action:** The proof from the manuscript is given below:

**Reviewer#1, Concern#4:** A reference should be provided in the list of references for the version of COMSOL used.

**Author response:** The authors are thankful to the honorable reviewer for their input. The reference of COMSOL is added to the revised manuscript.

**Author action:** The proof from the manuscript is given below:

**Reviewer#1, Concern#5:** The accuracy of the FEM-based models used must be verified by means of the results of appropriate FE mesh independence tests. Without these tests the FEM-based model can be regarded as incomplete. How to conduct these tests can be found in some papers recently published.

**Author response:** We agree with the honorable reviewer and have updated the revised manuscript accordingly. We sincerely thank the reviewer for their valuable efforts in improving the quality of our manuscript. This comment has indeed strengthened our work by leading us to include additional results and demonstrate the response of the FEM-based model through mesh independence testing.

**Author action:** The proof from the manuscript is given below:

**Reviewer#1, Concern#6:** The following sentence: "This leakage current can cause insulation failure and could become a major fault in the near future." is not based on research results and represents the Authors' opinion and should be modified accordingly.

**Author response:** We thank the honorable reviewer for highlighting this point. The statement is updated with reference.

**Author action:** The proof from the manuscript is given below:

**Reviewer#1, Concern#7:** Some abbreviations are defined more than once.

**Author response:** The authors are thankful to the reviewer for their in-depth study of the manuscript. The abbreviations defined more than once are corrected.

**Author action:** The proof from the manuscript is given below:

**Reviewer#1, Concern#8:** The grounding resistance is said to be assumed. Specifically, it says: "The grounding resistance is assumed to be 0.294534 Ohm". How was this value selected or estimated?

**Author response:** We thank the reviewer for this valuable observation. The grounding resistance value of 0.294534 Ω was selected as a representative and conservative estimate based on reported ranges in the literature for underground power systems. Following are the references noting a grounding resistance of 0.1 to 5 ohms.

[1] R. Araneo, G. Lovat, "Estimation of Substation Earth Impedance in Urban Networks," CIRED Open Access Proceedings Journal, vol. 2017, no. 1, pp. 1337–1340, 2017.
[3] IEEE Std. 80-2013, IEEE Guide for Safety in AC Substation Grounding, IEEE Power & Energy Society, 2013.

**Author action:** No update.

**Reviewer#1, Concern#9:** The conclusions must be improved in terms of scientific significance and quantified additionally based on the obtained results.

**Author response:** We agree with the honorable reviewer and updated the manuscript accordingly.

**Author action:** The proof from the manuscript is given below:

**Reviewer#2, Concern#1:** The magnetic field sensing equipment mentioned in the abstract is only validated through simulations in this paper, with no experimental verification using actual devices. Additionally, there is insufficient explanation of the equipment used in the study.

**Author response:** The authors agreed with the honorable reviewer and experimental validation of the proposed method is provided in the revised manuscript.

**Author action:** The proof from the manuscript is given below:

**Reviewer#2, Concern#2:** The use of abbreviations throughout the paper is inconsistent and confusing.

**Author response:** The authors are thankful to the reviewer for their in-depth study of the manuscript. The abbreviations defined are corrected.

**Author action:** The proof from the manuscript is given below:

**Reviewer#2, Concern#3:** The method proposed in this paper lacks comparative experiments, making it difficult to directly demonstrate the algorithm’s time complexity, power consumption, and the high cost associated with the large number of devices required.

**Author response:** The authors are grateful for the reviewer’s efforts in improving the quality of our manuscript. We agree with the honorable reviewer that comparative experiments with existing methods would provide a more direct demonstration of the superiority of the proposed method in terms of time complexity, power consumption, and device cost. However, the main objective of this study was to establish and verify the feasibility of the proposed non-invasive magnetic field sensing method. Therefore, a direct comparison was not included at this stage. Nonetheless, the cost-effectiveness of the proposed method can be inferred from the fact that it requires only a single movable magnetic sensor that is practically demonstrated in the **Experimental Validation** section added to the revised manuscript, in contrast to existing approaches that rely on multiple fixed sensors, thereby ultimately reducing computational effort, power consumption, and equipment costs.

**Author action:** The proof from the manuscript is given below:

**Reviewer#2, Concern#4:** The line charts used in Figures 20 and 21 are not suitable for effectively representing the specific errors. It is recommended to replace them with other types of graphs for better clarity.

**Author response:** The authors would like to thank the honorable reviewer for this valuable comment. The Figures 20 and 21 are replaced for better clarity.

**Author action:** The proof from the manuscript is give below:

**Reviewer#2, Concern#5:** The fault points described in the paper are at 100m, 500m, and 1000m. If all three points were to fail simultaneously, it is unclear whether the proposed method can accurately locate the faults. Further clarification or testing on this scenario would be necessary.

**Author response:** The authors deeply appreciate the honorable reviewer’s insightful comments and clarification on multiple fault points localization is added to the revised manuscript.

If fault occurs at multiple points on the same cable, the proposed method can accurately locate them one by one in the case of LGF and LLF only, starting from the fault point nearest to the supply. After clearing the first fault point, it is able to locate the next one, and then the third. However, locating all three points simultaneously is not possible with the proposed method.

**Author action:** The proof from the manuscript is given below:

**Reviewer#2, Concern#6:** In the paper, the tables present the values of Xact and Xcal along with their corresponding errors. However, the percentage error does not effectively reflect the precision of the proposed method. Additionally, there is a lack of comparative algorithms and their results, making it difficult to assess the effectiveness of the proposed algorithm.

**Author response:** We agree with the honorable reviewer that the percentage error primarily reflects the accuracy of the method rather than its precision. Nevertheless, the consistently low and stable error values across different fault locations demonstrate the precision and robustness of the proposed method. Comparative results with other algorithms were not included in the present study, however, we acknowledge this as an important direction for future work to further highlight the advantages of the proposed approach. In addition, experimental verification of the proposed method has been incorporated into the revised manuscript.

**Author action:** The proof from the manuscript is given below:

**Reviewer#3, Concern#1:**  In introduction part, the authors stated that the proposed method is cost effective. However, there is no verification on this statement.

**Author response:** The authors agree with the reviewer, and a clarification has been added in the revised manuscript. Specifically, the proposed method employs a single magnetic field sensing device that is moved across multiple measurement points, in contrast to existing methods that require several fixed sensors at different locations.

**Author action:** The proof from the manuscript is given below:

**Reviewer#3, Concern#2:** The paper is heavy on theoretical analysis but short on practice. How to measure the magnetic field? What kind of sensor is applicable to achieve magnetic field sensing for fault location? The impacts of environmental interferences (noises, electromagnetic, sampling interferences, etc.) should be considered.

**Author response:** We are thankful to the reviewer for this comment. The manuscript is updated accordingly.

**Author action:** The proof from the manuscript is given below:

**Reviewer#3, Concern#3:** What are the differences between LGF and LCF? The detailed fault developing mechanisms for the two types of faults are required.

**Author response:** We appreciate the reviewer’s efforts in helping to improve our manuscript. The manuscript is updated accordingly. The LCF is defined in detail to differentiate it from the LGF while the developing mechanisms of both the faults are discussed.

**Author action:** The proof from the manuscript is given below:

**Reviewer#3, Concern#4:**  In (2) and (13), the fault resistance Rg commonly vary with fault conditions. The reason why it is set as a constant should be explained.

**Author response:** The authors agree with the honorable reviewer that Rg varies with fault conditions. However, since Rg can be measured using established techniques such as the Fall-of-Potential (three-terminal) method and the Wenner method, it is considered constant in this study for the sake of simplicity and to focus on the applicability of the proposed method.

**Author action:** The proof from the manuscript is given below:

**Reviewer#3, Concern#5:**  Why magnetic field is used? Why not measure current or voltage signals? There are many sensors for electrical signals in practice but few sensors for field quantities. A comparison work is needed.

**Author response:** The authors thank the honorable reviewer for raising this important point. The primary reason for using magnetic field sensing instead of measuring current or voltage signals lies in the non-invasive nature of the approach. Conventional fault localization methods based on current and voltage require direct electrical connection to the cable, which is time consuming and laborious. In contrast, magnetic field sensing can be performed externally without physically accessing the conductor, making it more practical for buried cables. Moreover, the proposed method also determines the current, but instead of measuring it directly through sensors, it is calculated from the measured magnetic field, thereby avoiding the need for direct access to the cable.

**Author action:** No update.

**Reviewer#3, Concern#6:**  COMSOL is used to conduct the simulations. However, it is just an offline simulation tool. The reason why not use field data should be explained.

**Author response:** The authors thank the honorable reviewer for their valuable comment. The authors fully agree that field data would further strengthen the validity of the proposed method. However, at this stage, COMSOL Multiphysics was employed as it provides a controlled simulation environment where parameters such as burial depth, fault conditions, etc. can be systematically varied. This allows us to first establish the feasibility and accuracy of the method under different scenarios before moving to costly and logistically challenging field trials.

In addition, preliminary experiments were conducted in the campus laboratory to complement the simulation results and validate the methodology under practical conditions. Field-scale implementation is indeed the next step in our research plan, and we acknowledge this as an important direction for future work.

**Author action:** The proof from manuscript is given below:

***Note:*** *References suggested by reviewers should only be added if it is relevant to the article and makes it more complete. Excessive cases of recommending non-relevant articles should be reported to ieeeaccesseic@ieee.org*
