# Supplementary material for: Magnetic field sensing for 2D fault localization in underground power cables
Source: PLoS One. 2025 Oct 16;20(10):e0334515. doi: 10.1371/journal.pone.0334515 (PMC12530618; doi:10.1371/journal.pone.0334515)
Supplement: Attachment — Submitted filename: Response to reviewers 2.docx [file pone.0334515.s002.docx]

**Original Manuscript ID:** PONE-D-25-36102R1

**Original Article Title: “**Magnetic Field Sensing for 2D Fault Localization in Underground Power Cables”

**To:** Editor Plos One

**Re:** Response to reviewers

Dear Editor,

Thank you for allowing a revision of our manuscript, with an opportunity to address the reviewers’ comments.

We are uploading (a) our point-by-point response to the comments (below) (Response to Reviewers), (b) an updated manuscript with blue text indicating changes (as “Revised Manuscript with Track Changes*”*), and (c) a clean updated manuscript without highlights (“Manuscript”*).*

Best regards,

Hamid et al.

**Reviewer#1, Concern # 1:**  The following sentence "The experimental setup for LLF localization using the proposed magnetic field sending method is shown in Fig. 25." should be corrected.

**Author response:** The authors agree with the honorable reviewer and did as recommend.

**Author action:** The proof from the manuscript is given below:

**Reviewer#1, Concern # 2:** The results of FE mesh independence tests do not verify the accuracy of the proposed method. Authors are requested to perform these tests using papers published in the following journals: Applied Thermal Engineering, Electrical Engineering, Thermal Science, etc.

**Author response:** The authors have updated the FE mesh independence tests in the revised manuscript.

**Author action:** The proof from the manuscript is given below:

**Reviewer#1, Concern # 3:** In the following sentence "In these studies, the magnetic flux density of live cables was utilized to determine both the horizontal position and the vertical depth of the cable.", the term "live cables" should be replaced with any other more convenient term.

**Author response:** We agree with the honorable reviewer and updated the revised manuscript accordingly.

**Author action:** The proof from the manuscript is given below:

***Note:*** *References suggested by reviewers should only be added if it is relevant to the article and makes it more complete. Excessive cases of recommending non-relevant articles should be reported to ieeeaccesseic@ieee.org*
